# Intracranial Rosai-Dorfman Disease

**DOI:** 10.1155/2014/724379

**Published:** 2014-02-11

**Authors:** Yadav Arun Kumar, Peng Yi Peng, Xia Chen Chen

**Affiliations:** Department of Radiology and Imaging, Shanghai 10th People's Hospital, Tongji University, Yanchang Road, Building No. 301, Shanghai 200072, China

## Abstract

Rosai-Dorfman disease (RDD) is a rare, benign pseudolymphomatous condition, predominantly involving lymph nodes. Rosai-Dorfman disease (RDD) (sinus histiocytes with massive lymphadenopathy) rarely affects the intracranial region without involvement of other sites. It is a rare and idiopathic histoproliferation disorder characterized by painless lymphadenopathy. We report a case of 43-year-old male who presented with unconsciousness; MRI was done and right temporofrontal mass was found. Excision was done, and on histopathology it confirmed RDD.

## 1. Introduction

Rosai-Dorfman disease is a benign lymphohistiocytosis that often involves lymph nodes and presents as massive lymphadenopathy with sinus histocytiosis characterized by painless cervical lymphadenopathy, fever, leukocytosis, elevated ESR and polyclonal hypergammaglobulinemia. The disease was first described by Destombes in 1965 and later, in 1969, Rosai-Dorfman disease was first reported by Juan Rosai and Roland Dorfman as sinus histiocytosis with massive lymphadenopathy in young black males. RDD predominantly affected the children and young adult with mean age of 20.6 years but can be seen in the range of 1 to 74 years old. There is a slight male to female predominance (male : female = 1.4 : 1) [1]. The central nervous system can be involved in less than 5% [2]. Over 90% of patients present with cervical lymphadenopathy, and extranodal involvement including paranasal sinuses, skin, bone and Orbit which are seen in 43% of cases [3].

## 2. Case Report

43-year-old male was referred to our hospital in December 2012, with history of new onset of sudden fainting attack at home for 8 min 4 days ago. He was unconscious for 8 min but with no seizure, no mouth frothing, no uprolling of eyes, no headache, no nausea, vomiting. At local hospital he has done MRI and found right temporofrontal lobe mass with edema. Four days later he came to our hospital in outpatient department of neurosurgery for further checkup. On the time of arrival in our hospital he was fully conscious and well oriented; GCS 15/15. He has never experienced this symptom before. There was no significant past history, no family history; hearts and lungs are clear. Vitals are within normal limit. Patient had normal movement, symmetrical pupil size, normal head wrinkle, normal facial expression and lymph node not palpable. On abdomen, spider nevi sign was seen. Muscle power was 5/5. Pathological sign was negative. He is smoker and consumes alcohol occasionally. MRI (Siemens 3.0 T) with contrast was repeated and showed right temoporofrontal lobe mass, subdural mass, corona radiate edema, and B/L sinusitis. Differential diagnosis was made on meningioma and glioma. EEG was unremarkable. CT scan was performed and showed left lower lung nodule and chronic inflammation. Partial removal of right temporofrontal mass was done and 5.5 × 4 × 2.5 tissue size was found, which was grey yellow in color. Sample was sent for histopathology, and Rosai-Dorfman disease was confirmed. The rest blood test included ESR, WBC, CRP, TC, DC, and HB which were normal. Urine test and tumor markers were normal such as AFP, Ca199, and Ca152 except CEA which was found as high as 8.35 (normal: 5). Histopathology reported that S-100 protein was found positive and CD*α* was found negative.

## 3. Discussion

Rosai-Dorfman disease rarely occurs in CNS. Most of time it occurs in cervical lymph node. Postulated causes include infectious causes, immunodeficiency, autoimmune disease, and a neoplastic process; however, none has been substantiated [4]. Regarding CNS RDD, the most frequent locations include the cerebral convexities, the parasagittal, suprasellar, cavernous sinuses, and the petroclival regions. Patients with intracranial involvement usually present with headache and seizures. They can also present with dysphasia, cranial nerve deficit, progressive loss of vision, hemiparesis, neglect, and endocrine dysfunction depending on the location of the lesion [5]. In our case, patient has new onset of unconsciousness, but there are no headache, no cervical lymphadenopathy, and no seizures, no ESR elevated; this is atypical intracranial Rosai-Dorfman disease. CNS can be involved in less than 5% of Rosai-Dorfman disease. In 70% of CNS-Rosai-Dorfman disease, the presentation is limited to the brain or to the spinal cord and is not associated with lymphadenopathy [2]. Most intracranial RDD lesions appear to be radiologically mimicking meningioma. According to Kumar et al., comparing plain X-ray film radiography in case of meningioma with RDD, it is seen that hyperostosis, erosion, tumor calcification, and enlarged vascular channels are not present in RDD. On CT, generally RDD mass lesion shows hyperdense and enhancement on contrast as seen in meningioma. Magnetic resonance imaging findings in case of RDD mostly mimic the meningioma [3]. The RDD mass is mostly isointense on T1- and T2-weighted images with strong contrast enhancement dural tail being uncommon. It is difficult to distinguish RDD from meningioma radiologically. In our case as shown in Figure 2, (a) TIWI image shows slightly hypointense lesion on Rrt. Temoral region with slight midline shift. (b) T2WI shows hyperintense mass by edema suttounding with midline shift. (c) TIWI C+, after surgery, shows isointense lesion. (d) DWI: diffusion weighted image hyperintense lesion with midline shift. Perilesional oedema is less prominent in case of RDD as compared to meningioma [3]. The histopathologic differential diagnosis of this particular case included lymphoma, plasma cell granuloma, plasma cell rich meningioma, and Langerhans cell histiocytosis.

**Figure 1:**
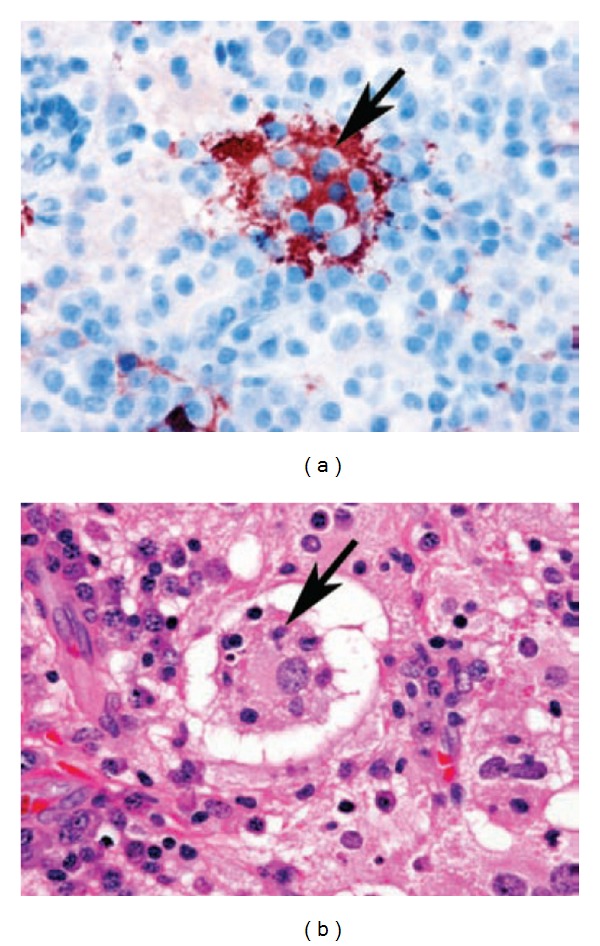
(a), (b) [6]. Histiocytic cells with emperipolesis are immunoreactive for S-100 protein and negative for EMA. (a) Photomicrograph (H and E, original magnification, ×400) shows large histiocytes that display prominent intracytoplasmic lymphocytes (*arrow*). (b) Photomicrograph (S100 immunostaining, original magnification, ×400) shows diffuse, strong cytoplasmic positivity within histiocytes (*arrow*). Emperipolesis, that is, engulfment by Rosai-Dorfman disease histiocytes of lymphocytes and other reactive inflammatory cells is evident in this image (a).

**Figure 2:**
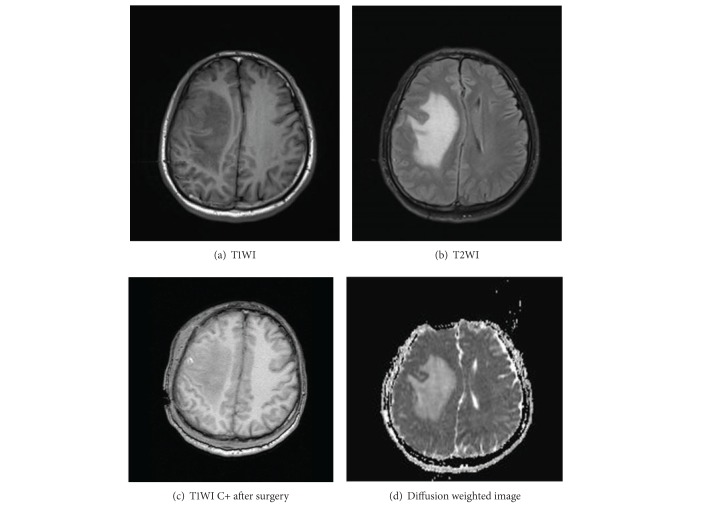
(a) T1WI images shows slightly hypointense lesion on Rt. temporofrontal region with slight midline shift. (b) T2WI shows hyperintense mass by edema surrounded with midline shift. (c) T1WI C+, after surgery, shows isointense lesion. (d) DWI: diffusion weighted image hyperintense lesions with midline shift.

Histopathologically, the Rosai-Dorfman disease infiltrate shows “sinusal” lymph node architecture with clustering of lymphocytes simulating the appearance of germinal centers. The characteristic histopathologic feature is emperipolesis Figure 1, in which histiocytes, phagocyte, lymphocytes, plasma cells, erythrocytes, or polymorphonuclear leukocytes are intact, this is more appropriate [2–6]. Immunohistochemical analysis consistently shows S-100 protein positivity, particularly with the monoclonal antibody and immunoreactivity against *α*-1-antichymotrypsin, CD68, EMA, and MAC387 antibodies [2–7]. The characteristic immunohistochemistry features are positive S-100 protein, Ki67 5%, CD79*α*, and LCA with negative CD1*α*, CK, EMA and GFAP shows in Table 1. According to Konishi, the prognosis of Rosai-Dorfman disease with involvement of the CNS was not poor. In a review of follow-up data of 43 patients, most patients (58%) were alive with disease. Only two patients (4.7%) had died. Moreover, no death was reported to have occurred as a result of isolated intracranial Rosai-Dorfman disease [8].

Multiple strategies of therapy have been used with varying success, including radiation therapy, chemotherapy, steroids, and surgery [4]. Surgical resection seems to be the most effective treatment. Recurrence after surgical therapy is rare and limited to uncompleted debulking, multiorgan involvement, and large mass [2]. After surgery usually 1000 CGy is given in 200 CGy daily doses. Few reports showed resolution with corticosteroids and chemotherapy. Potential treatment in the future includes 2-chlorodeoxyadenosine (2CDA), monoclonal antibody targeting with indium labelled anti-CD1a [3]. Prognosis of the disease is good. The most effective therapeutic regimen would be a combination of corticosteroid (prednisolone) and vinca alkaloids (vincristine and vinblastine) with an alkylating agent (cyclophosphamide) [7].

## 4. Conclusion

Rosai-Dorfman disease is benign. Although Rosai-Dorfman disease is a rare process, we present a case of extranodal RDD involving Rt. Temporo-parietal intracranial region. It should be included in the differential diagnoses for a dural mass mimicking meningioma or cerebral mass mimicking glioma. It can be misinterpreted as plasma cell granuloma, plasma cell rich meningioma, and Langerhans cell histiocytosis in histopathological examinations due to an intense infiltration of inflammatory cells. Thus, a definite diagnosis relies on the histological pattern and immunohistochemical characterization of the lesions. Therefore, immunohistochemical staining for EMA, S100, and CD1a should be performed. Surgical excision of the lesion is the treatment of choice. In cases with subtotal tumor resection or recurrence of the lesion, adjuvant therapy with local low dose radiotherapy and steroids can be considered. Prognosis is benign especially in the absence of nodal disease.

## Figures and Tables

**Table 1:** Immunohistochemistry report of our case.

S-protein	Positive
CD1*α*	Negative
CK	Negative
EMA	Negative
GFAP	Negative
LCA	Positive
Part of CD20	Positive
CD79*α*	Positive
CD	Partially positive
CD4	Partially positive
CD5	Slightly positive
CD8	Positive
BCL-2	Positive
CD34 in vessels	Positive
Ki67 5%	Positive

## Key Message

The clinical presentation, differential diagnosis, and treatment modality of RDD are discussed here.
